# Supplementary material for: Molecular Re-Diagnosis with Whole-Exome Sequencing Increases the Diagnostic Yield in Patients with Non-Syndromic Retinitis Pigmentosa
Source: Diagnostics (Basel). 2023 Feb 14;13(4):730. doi: 10.3390/diagnostics13040730 (PMC9954991; doi:10.3390/diagnostics13040730)
Supplement: Supplementary file 1 [file diagnostics-13-00730-s001.zip › diagnostics-2189491-supplementary.pdf]

Table S1 Oligonucleotide primers used to perform copy number analysis

Primer	Sequence (5’ to 3’, Hg19)	Genomic coordinates	Target
Reference genes			
ALB_conF	TGAAATGGCTGACTGCTGTG	chr4:74274367-74274449	Autosomal reference *ALB*
ALB_conR	GGAGGTTTGGGTTGTCATCT		
F8_conF	TTTCCATTCAACACCTCAGTCGT	chrX:154227766-154227850	X chromosome reference *F8*
F8_conR	GCCTTGGCTTAGCGATGTTG		
Family F5, index patient S5			
OSCARint3F	AACCAGGTGCAAATCAGAGG	chr19:54600621-54600710	Normal 5’ region
OSCARint3R	TTCTGGGACCTGACCTTTTG		
TFPTint1F	CAAGAGTCCTGGCTTCCAAC	chr19:54618400-54618494	Deleted region at the 19q13.42 *locus*
TFPTint1R	CCTTTTATGGACTGGGGTCA		
PRPF31ex6F	CCTGGACAAGTGCAAGAACA	chr19:54626847-54626921	
PRPF31ex6F	GTGACGCTGACGACCATGA		
PRPF31ex14F	GAGGAGGCCTTGGAAGAGTC	chr19:54634994-54635080	
PRPF31ex14R	AGGCATGATGAAGACCTGCT		
19q13.42_AF	CTCTGGACCTCAGTGGCAAT	chr19:54638212-54638306	Normal 3’ region
19q13.42_AR	GGGGTAGTTCAGCCACTCAA		
Family F9, index patient S9			
USH2Aint24F	AAACTAAGCTTTGCCCCCATA	chr1:216258815-216258885	Normal 5’ region
USH2Aint24R	AGGAGGAGAAGGTGCACTGA		
USH2Aex24F	GTTCCTCTGCCATCCTGAAT	chr1:216260094-216260162	Deleted region in the *USH2A* gene
USH2Aex24R	GGAGCCCTCCCAGAAAGAC		
USH2Aex23F2	GGGGTCACCAGTGGAAGTAA	chr1:216262384-216262482	
USH2Aex23R2	CCAAAAGCCTGATGCCTAAT		
USH2Aex22F	CAAAAGTGCCTGAAGGTTTGA	chr1:216270445-216270534	
USH2Aex22R	AAAGACGTCCCTTCTTCAACTG		
USH2Aint21F	GTGCAGGTTGACCCATTTTT	chr1:216276541	

USH2Aint21R	GGGGGCCCAGTAGTGTTCTA	-216276623	
USH2Aint21F6	TCATCTCTCCCACCAAGGAC	chr1:216339631 -216339722	Normal 3’ region
USH2Aint21R6	TGGGCATCTGAGAGAGTGTG		
Family F12, index patient S12			
qEYSint28_4F	AGACCCTGCATGGTTGTGTT	chr6:65134995-65135079	Normal 5’ region
qEYSint28_4R	AAGTGAATGCAGCGTGTGAG		
qEYSint27F	GCATGCTCTTTTCCCAACAT	chr6:65147921-65148004	Duplicated region in the *EYS* gene
qEYSint27R	GGGATGAGTTCCCTAAACCA		
qEYSex27F	TCCACAAAATAACATCTCCCTAGA	chr6:65149105-65149184	
qEYSex27R	AATTTGAGTCTTGCTTGACATACA		
qEYSint26_2F	ATTCTCATGCAAGGGGAGTG	chr6:65156074-65156156	
qEYSint26_2R	TGCTAGTTGCCAGCTTTTATTT		
qEYSint26_3F	AATGTAGGGACCTGGCATTG	chr6:65169892-65169971	Normal 3’ region
qEYSint26_3R	TTGTGCCAGGACTCTTTCCT		
Family F15, index patient F15			
PRPF31ex2int2F	TCGCCAAGCTATGGGATAGT	chr19:54621810 -54621906	Normal 5’ region
PRPF31ex2int2R	AGCCTGTATCACCCCCTTCT		
PRPF31int4ex5F	GCCAACCAGCAGAGTCTACC	chr19:54625816 -54625899	Deleted region in the *PRFP31* gene
PRPF31int4ex5R	CCCGGATGAACTTATGGATG		
PRPF31ex6F	CCTGGACAAGTGCAAGAACA	chr19:54626847 -54626921	
PRPF31ex6F	GTGACGCTGACGACCATGA		
PRPF31ex7F	CTCCAAGCACCGCATCTA	chr19:54627197 -54627272	
PRPF31ex7F	CCCGATAATGATGGACAGGT		
PRPF31ex8F	CCTGACCAACCTCTCCAAGA	chr19:54627891 -54627974	Normal 3’ region
PRPF31ex8R	GTAGACGAGAAGCCCGACAG		

Table S2 qPCR, long-range and sequencing oligonucleotide primers used to establish breakpoints of the CNVs

Primer*	Sequence (5' to 3', Hg19)	Genomic coordinates	Index patient
NDUFA3int2F	TCTTTCCTCTCCCCCATTCT	chr19:54606793-54606884	S5
NDUFA3int2R	ATTGGGATCATACGGGGATT		
19q13.42_DF	GCTGGCCTCCTGGTTAATTT	chr19:54635648-54635740	
19q13.42_DR	GCCAAGATTGCACCACTGTA		
19q13.42_EF	TGTTGAGGGACTGTGTGGAG	chr19:54636703-54636813	
19q13.42_ER	GGAAGTGTGCTCCTTCTGCT		
USH2Aint21_F7	TACCCCTTCTTACCGCATTG	chr1:216318637-216318718	S9
USH2Aint21_R7	GTACAGCAAGGGCCAAGGTA		
USH2Aint21_F8	CTGGGGAAGTCTTGCTGAAC	chr1:216323893-216324000	
USH2Aint21_R8	CAACATCTCCCGTCTCCCTA		
qEYSint28_8F	CTTGACCTTGGGTGATCCTC	chr6:65135807-65135949	S12
qEYSint28_8R	GAAGCAGCCAGGTGTGGT		
qEYSint28_9F	TTTTTATGCCTGTTCCAGCA	chr6:65135807-65135949	
qEYSint28_9R	AGGAGTAGGAGACCAGGAATTG		
qEYSint26_6F	TGAAAAGTGTCCCCACACAA	chr6:65164485-65164567	
qEYSint26_6R	CAACAAAATGCCAACCAAAA		
qEYSint26_9F	GGCTCATGCCTGCCTATAAT	chr6:65164983-65165095	
qEYSint26_9R	TGTGTGGAGACAGGATCTCG		
PRPF31int3F	TCTCACAAGCTGTGGCAGTC	chr19:54623728-54623815	S15
PRPF31int3R	TCCCTTTCTGGAAGGATCAG		
PRPF31int3F2	GAGGCCCTGAATGTCAGTCT	chr19:54624937-54625021	
PRPF31int3R2	CCAGAACTGACCGTGTGAAA		

* The sequencing primers used in cases S5, S9 and S15 are underlined

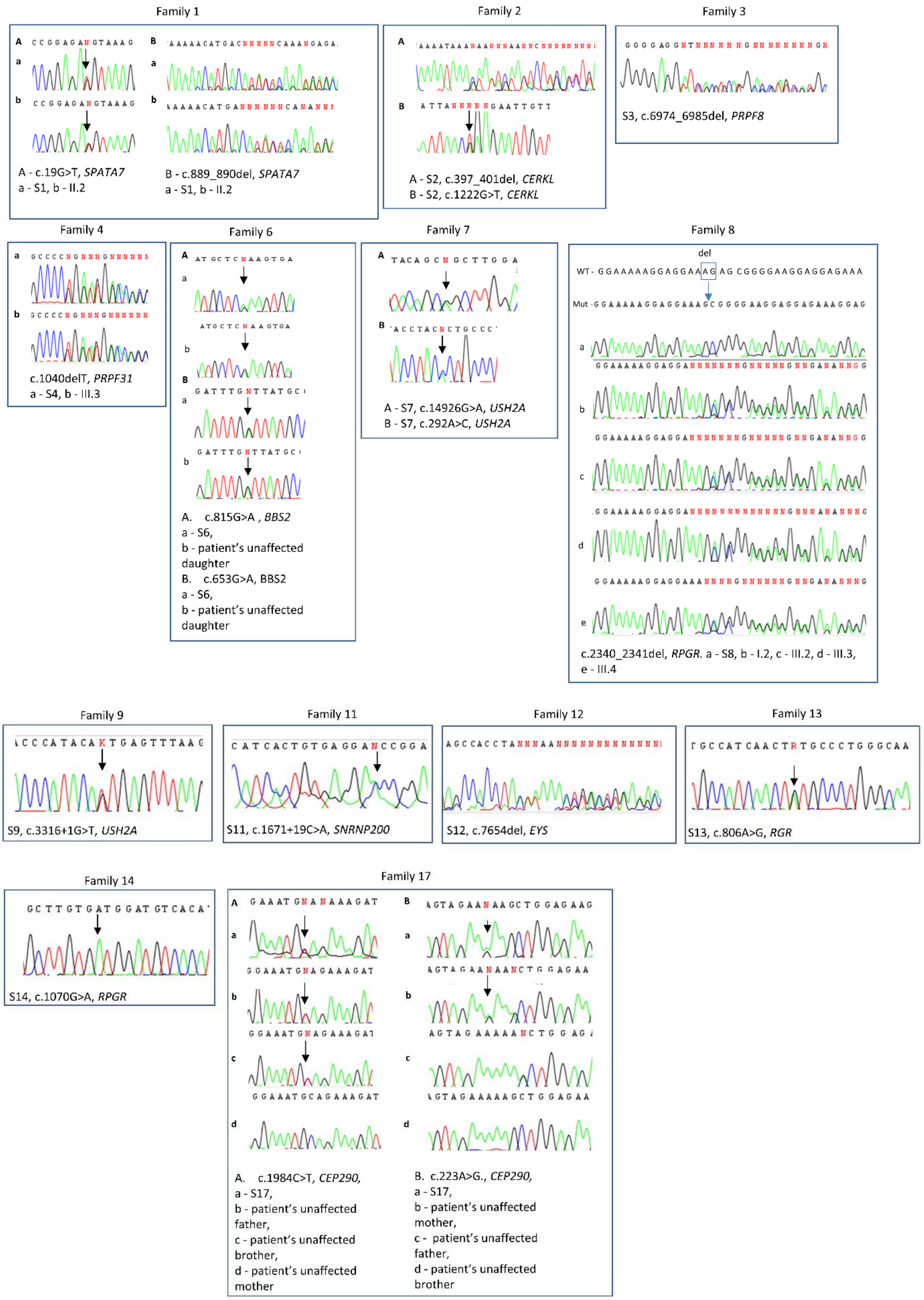
Family 1
A - c.19G>T, SPATA7
a - S1, b - II.2
B - c.889_890del, SPATA7
a - S1, b - II.2
Family 2
A - S2, c.397_401del, CERKL
B - S2, c.1222G>T, CERKL
Family 3
S3, c.6974_6985del, PRPF8
Family 4
c.1040delT, PRPF31
a - S4, b - III.3
Family 6
A. c.815G>A , BBS2
a - S6,
b - patient's unaffected daughter
B. c.653G>A, BBS2
a - S6,
b - patient's unaffected daughter
Family 7
A - S7, c.14926G>A, USH2A
B - S7, c.292A>C, USH2A
Family 8
del
c.2340_2341del, RPGR. a - S8, b - I.2, c - III.2, d - III.3, e - III.4
Family 9
S9, c.3316+1G>T, USH2A
Family 11
S11, c.1671+19C>A, SNRNP200
Family 12
S12, c.7654del, EYS
Family 13
S13, c.806A>G, RGR
Family 14
S14, c.1070G>A, RPGR
Family 17
A. c.1984C>T, CEP290,
a - S17,
b - patient's unaffected father,
c - patient's unaffected brother,
d - patient's unaffected mother
B. c.223A>G., CEP290,
a - S17,
b - patient's unaffected mother,
c - patient's unaffected father,
d - patient's unaffected brother

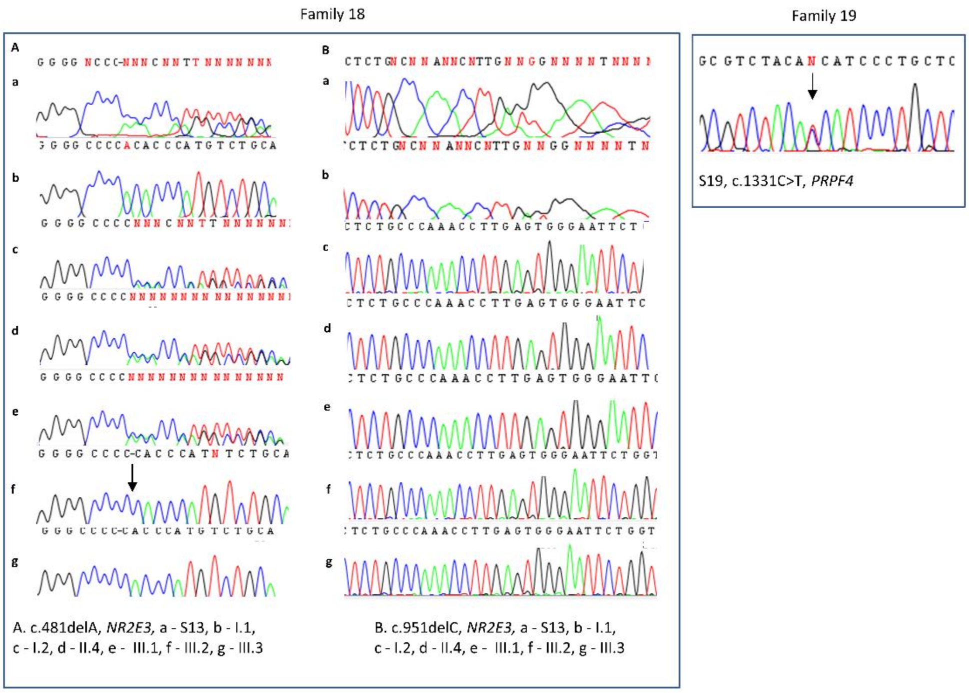

Figure S1 Sequencing results of the variants identified in RP patients and the segregation analysis in familial RP cases.

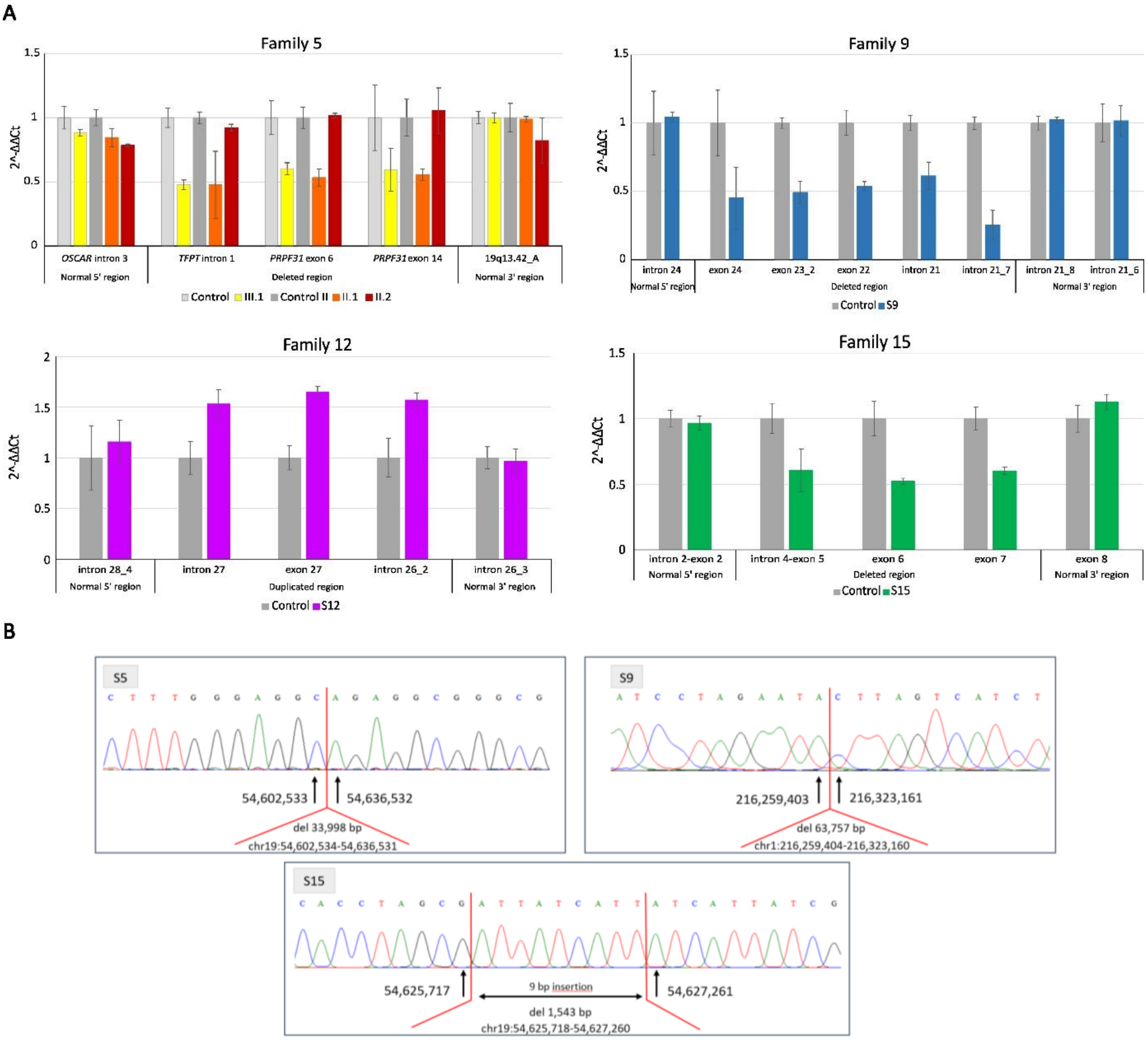

Figure S2 **qPCR validation and breakpoint sequencing of the CNVs**

qPCR results in families F5, F9, and F15 show lower copy number, tantamount to 1 copy, validating studied CNVs in all affected individuals, while the higher copy number, tantamount to 3 copies, was identified in family F12. Error bars represent standard deviation. Normalization was performed against the *ALB* gene (F5, F9, and F15) or the *F8* gene (F12). In family F5, the deletion in the 19q13.42 region, encompassing *PRPF31*, among other genes, was confirmed in the affected proband (III.1) and her father (II.1). The unaffected mother (III.2) was negative for the lesion. Following samples were run separately: control and III.1 (experiment I), control II, III.1, and III.2 (experiment II). In family F9, the deletion of exons 22-24 of the *USH2A* gene was detected in the affected proband (S9). In family F12, a duplication of a portion of the *EYS* gene, encompassing exons 27 and 28, was observed in the affected index. In family F15, the deletion of exons 5, 6, and partly 7 in the *PRPF31* gene was identified in the affected proband. The obtained results pointed to the heterozygous state of all aforementioned CNVs. (B) Chromatograms showing breakpoints of the deletions identified in families F5, F9, and F15.

Reference sequences were obtained from the UCSC Genome Browser on Human Feb. 2009 (GRCh37/hg19) Assembly.

Autosomal Dominant Retinitis Pigmentosa NGS panel, 26 genes

Genes : AIPL1, BEST1, CA4, CRX, FSCN2, GUCA1B, IMPDH1, KLHL7, NR2E3, NRL, PRKCG, PRPF3, PRPF6, PRPF8, PRPF31, PRPH2, RDH12, RGR, RHO, ROM1, RP1, RP9, RPE65, SEMA4A, SNRNP200, TOPORS

Autosomal Recessive Retinitis Pigmentosa, NGS panel, 56 genes

Genes: ABCA4, AIPL1, ARL6, BEST1, C2orf71, C8orf37, CA4, CERKL, CLRN1, CNGA1, CNGB1, CRB1, CRX, DHDDS, EYS, FAM161A, FLVCR1, FSCN2, GUCA1B, IDH3B, IMPDH1, IMPG2, LRAT, MAK, MERTK, NR2E3, NRL, PDE6A, PDE6B, PDE6G, PRCD, PROM1, PRPF3, PRPF6, PRPF8, PRPF31, PRPH2, RBP3, RDH12, RGR, RHO, RLBP1, ROM1, RP1, RP2, RP9, RPE65, RPGR (ORF15 excluded), SAG, SEMA4A, SPATA7, TOPORS, TTC8, TULP1, USH2A, ZNF513

Methods

Targeted next-generation sequencing

Sequencing was carried out on an Illumina NextSeq, a variant-discovery pipeline was built on the basis of GATK Best Practices. The human reference genome (hg19) was used. Filtering and interpretation of NGS data were done using Exomiser running the hiPHIVE algorithm. Variants were visualized using an Integrative Genomics Viewer (IGV; Broad Institute and the Regents of the University of California).

Sanger sequencing

The online Primer3Plus tool (version: 3.2.6) was used to design specific primers for amplification. The sequencing products were separated on an ABI 3130xl capillary sequencer (Applied Biosystems). Variants were annotated against the reference sequences: SPATA7 – NM_018418.5, CERKL – NM_201548.5, PRPF8 – NM_006445.4, PRPF31 – NM_015629.4, BBS2 – NM_031885.4, USH2A – NM_206933.3, RPGR – NM_001034853.2, RP1L1 – NM_178857.6, SNRNP200 – NM_014014.5, EYS – NM_001292009.1 , RGR – NM_002921, CEP290 – NM_025114.4, NR2E3 – NM_014249.4, PRPF4 – NM_004697.4 following the Human Genome Variation Society (HGVS) nomenclature guidelines.

Quantitative real-time polymerase chain reaction (qPCR)

SYBR Green PCR Master Mix (Applied Biosystems) was used and all reactions were run in in triplicate on a ViiA™ 7 thermal cycler (Applied Biosystems). ALB and F8 were used for normalization, the later

constituting an internal sample control (based on sex determination). For the analysis the comparative $2^{-\Delta\Delta CT}$ method was used, with noncommercial healthy control DNA as a calibrator.

Breakpoint sequencing

Standard DNA amplification reactions were set as described previously using the FailSafe™ PCR 2X Premix J (Epicentre). The PCR conditions were as follows: initial denaturation step at 95°C for 3 min followed by 40 cycles of denaturation at 94°C for 15 sec, annealing at 63°C for 45 sec, with temperature starting from 63°C, decreasing to 55°C (touchdown PCR −0.2°C per cycle), elongation at 72°C for 90 sec, with final elongation at 72°C for 10 min. The obtained PCR products were sequenced as described above.
